# Clarithromycin-Associated Acute Liver Failure Leading to Fatal, Massive Upper Gastrointestinal Hemorrhage from Profound Coagulopathy: Case Report and Systematic Literature Review

**DOI:** 10.1155/2020/2135239

**Published:** 2020-02-18

**Authors:** Ahmed I. Edhi, Seifeldin Hakim, Christienne Shams, Damanpreet Bedi, Mitual Amin, Mitchell S. Cappell

**Affiliations:** ^1^Division of Gastroenterology & Hepatology, Department of Medicine, William Beaumont Hospital, 3535 W Thirteen Mile Rd, Royal Oak, MI 48073, USA; ^2^Department of Medicine, William Beaumont Hospital, Oakland University William Beaumont School of Medicine, 3535 W Thirteen Mile Rd, Royal Oak, MI 48073, USA; ^3^Transplant Surgery, Department of Surgery, William Beaumont School of Medicine, 3601 W Thirteen Mile Rd, Royal Oak, MI 48073, USA; ^4^Department of Pathology, William Beaumont Hospital, Oakland University William Beaumont School of Medicine, 3601 W Thirteen Mile Rd, Royal Oak, MI 48073, USA; ^5^Division of Gastroenterology & Hepatology, Department of Medicine, Oakland University William Beaumont School of Medicine, 3535 W Thirteen Mile Rd, Royal Oak, MI 48073, USA

## Abstract

While erythromycin has caused numerous cases of acute liver failure (ALF), clarithromycin, a similar macrolide antibiotic, has caused only six reported cases of ALF. A new case of clarithromycin-associated ALF is reported with hepatic histopathology and exclusion of other etiologies by extensive workup, and the syndrome of clarithromycin-associated ALF is better characterized by systematic review. A 60-year-old nonalcoholic man, with normal baseline liver function tests, was admitted with diffuse abdominal pain and AST = 499 U/L and ALT = 539 U/L, six days after completing a 7-day course of clarithromycin 500 mg twice daily for suspected upper respiratory infection. AST and ALT each rose to about 1,000 U/L on day-2 of admission, and rose to ≥6,000 U/L on day-3, with development of severe hepatic encephalopathy and severe coagulopathy. Planned liver biopsy was cancelled due to coagulopathies. Extensive evaluation for infectious, immunologic, and metabolic causes of liver disease was negative. Abdominal computerized tomography and abdominal ultrasound with Doppler were unremarkable. The patient developed massive, acute upper gastrointestinal bleeding associated with coagulopathies. Esophagogastroduodenoscopy was planned after massive blood product transfusions, but the patient rapidly expired from hemorrhagic shock. Autopsy revealed a golden-brown heavy liver with massive hepatic necrosis and sinusoidal congestion. Rise of AST/ALT to about 1,000 U/L each was temporally incompatible with shock liver because this rise preceded the hemorrhagic shock, but the subsequent AST/ALT rise to ≥6,000 U/L each may have had a component of shock liver. The six previously reported cases were limited by failure to exclude hepatitis E (4), lack of liver biopsy (2), and uninterpretable liver biopsy (1) and by confounding potential etiologies including disulfiram, israpidine, or recent acetaminophen use (3), clarithromycin overdose (1), active alcohol use (1), and severe heart failure (1). Review of 6 previously reported and current case of clarithromycin-associated ALF revealed that patients had AST and ALT values in the thousands. Five patients died and 2 survived.

## 1. Introduction

Acute liver failure (ALF) refers to severe acute liver injury with encephalopathy and impaired liver synthetic function developing within 4 weeks in a patient with prior normal liver function or well-compensated liver disease [1]. ALF is commonly due to drug-induced liver injury (DILI), with implicated drugs in the United States including acetaminophen overdose (46%), idiosyncratic reactions to various drugs (12%), and occasionally (14% of cases) in which a drug is a possible cause of ALF of uncertain origin [2]. Clarithromycin is a macrolide antibiotic widely used to treat respiratory, skin, or soft tissue infections and infections by *Mycobacterium avium* complex or *Helicobacter pylori.*While erythromycin, a related macrolide antibiotic, has caused numerous cases of ALF [3, 4], comprehensive literature review revealed only 6 reported cases of clarithromycin-induced ALF [5–10], in addition to moderate liver injury with moderately elevated liver enzymes and cholestatic hepatitis ascribed to clarithromycin [11, 12]. A case of ALF secondary to clarithromycin is reported, and the syndrome is systematically reviewed.

## 2. Methods

Systematic literature search on liver injury from clarithromycin using PubMed and Medline with the following medical subject headings/keywords: [(“liver injury”) OR (“drug induced liver injury”) OR (“DILI”) OR (“acute hepatic failure”) OR (“fulminant liver failure”) OR (“acute liver failure”)] AND [(“clarithromycin”)] and by reviewing sections on DILI in medical, hepatology, or pathology textbooks. All articles, including case reports, were reviewed. Two authors independently performed literature searches, independently reviewed prior case reports, and reconciled differences to minimize errors. Two authors abstracted the case report from the electronic medical record and reconciled differences to reduce errors.

## 3. Case Report

A 60-year-old nonalcoholic man with no known liver disease who was chronically administered the following drugs: albuterol and budesonide-formoterol inhalers for asthma; levothyroxine 100 mcg once daily for hypothyroidism; and amlodipine 5 mg once daily, metoprolol 25 mg once daily, and lisinopril 20 mg twice daily for hypertension, presented with atypical chest pain, dyspnea, and productive cough. Physical examination revealed normal vital signs, bilateral wheezing in both lung fields, and no hepatosplenomegaly or stigmata of chronic liver disease. Serial cardiac enzymes and electrocardiograms were within normal limits. Chest roentgenogram revealed no pulmonic consolidation or infiltrates. Laboratory analysis revealed leukocytosis, normal hemoglobin and platelet counts, and normal blood urea nitrogen and creatinine levels. Liver function tests were initially normal, except for an alanine aminotransferase (ALT) level of 72 U/L (Table 1, column 2). He was diagnosed with asthma exacerbation secondary to upper respiratory tract infection and treated with clarithromycin 500 mg twice daily orally for 7 days; intravenous corticosteroids, which were subsequently converted to a tapering dose of oral prednisone; and resumption of home medications. He was discharged on hospital day 6 when his symptoms subsided after completing 3 days of clarithromycin therapy.

Ten days after discharge and 6 days after completing a seven-day course of clarithromycin, the patient represented with diffuse abdominal pain for 4 days. Physical examination revealed blood pressure of 147/92 mmHg; otherwise normal vital signs; a soft, distended, diffusely tender abdomen with hypoactive bowel sounds and no rebound tenderness or guarding; and no stigmata of chronic liver disease. There was mild leukocytosis and normal hemoglobin level, while the aminotransferase levels were significantly elevated, and international normalized ratio (INR) was 0.9 (Table 1, column 3). The lactic acid was 0.6 mmol/L (normal: 0.5–2.2 mmol/L), ammonia was 22 *µ*mol/L (normal 11–35 *µ*mol/L), and acetaminophen level was <4.0 *µ*g/ml. The next day vital signs were stable, with no change in abdominal exam. The AST and ALT levels rose to >1,000 U/L, and lactate dehydrogenase was 4,200 U/L. Extensive tests for viral hepatitis, including anti-HAV, anti-HBs, anti-HBc, HBs antigen, anti-HCV, HCV-RNA, and anti-HEV, were negative, as were tests for human immunodeficiency virus, cytomegalovirus, and Herpes simplex virus. Blood cultures were sterile. The autoimmune hepatitis panel, including anti-nuclear antibody (ANA), anti-mitochondrial antibody (AMA), anti-smooth muscle antibody (ASMA), anti-liver-kidney microsomal (LKM) antibodies, and total immunoglobulin, was negative or within normal limits. Tests for genetic hemochromatosis were negative. Ceruloplasmin level was normal. Slit-lamp ophthalmologic examination did not reveal Kayser-Fleischer rings. Abdominal computerized tomography (CT) with IV contrast and abdominal ultrasound with Doppler studies were unremarkable. Echocardiography was normal.

On day 3, the patient was transferred to an intensive care unit because of altered mental status, asterixis, and ammonia of 96 *µ*mol/L, findings consistent with grade III hepatic encephalopathy. The AST and ALT rose to ≥6,000 U/L (Table 1, column 5), and the hemoglobin declined to 5.5 g/dL, with severe melena from acute upper gastrointestinal bleeding and development of borderline hypotension (Supplementary [Supplementary-material supplementary-material-1]). Bleeding was associated with 35,000 platelets/*µ*L, prothrombin time >100 seconds, activated partial thromboplastin time >124 seconds, D-dimer >9,999 ng/ml, and fibrinogen <35 mg/dL. Massive blood products were transfused. EGD, planned because of acute melena, and liver biopsy, planned for worsening liver failure, were both postponed due to hemodynamic instability and coagulopathy. Despite aggressive resuscitation, the patient rapidly deteriorated, suffered cardiac arrest, and expired.

**Figure 1:**
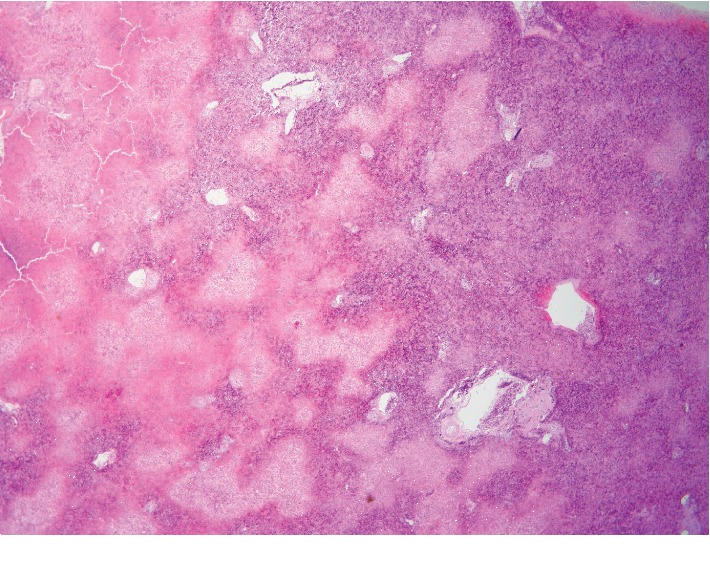
Low power photomicrograph of hematoxylin and eosin stain of liver section obtained at autopsy shows geographic areas of necrosis and marked sinusoidal congestion.

**Figure 2:**
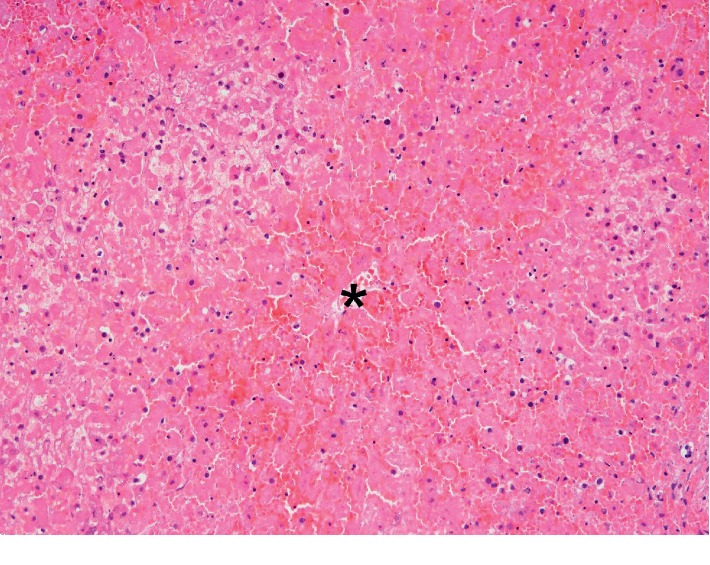
High power photomicrograph of hematoxylin and eosin stain of liver section obtained at autopsy shows a central vein (∗) with adjacent marked sinusoidal congestion and extensive hepatocyte necrosis.

The family consented to autopsy confined to liver, kidneys, lungs, and pancreas. Liver postmortem examination grossly revealed a golden-brown soft liver weighing 1,980 gm. Histopathologic hepatic examination revealed massive hepatocyte necrosis, occasional sparing of periportal hepatocytes, and diffuse sinusoidal congestion (Figures 1 and 2). No lobular or portal inflammation, fibrosis, viral cytopathic effect, thromboemboli, or malignancy was evident. Immunohistochemistry revealed no Herpes simplex virus types 1 and 2 or cytomegalovirus.

## 4. Discussion

DILI accounts for approximately 10% of acute hepatitis, but is the most common cause of ALF in the United States and the most frequent reason for drug withdrawal from the marketplace [13]. DILI is classified as predictable, as for acetaminophen, or idiosyncratic, as for amoxicillin/clavulanate. DILI is also classified according to the liver injury pattern as (1) hepatocellular (primarily elevated ALT and AST), (2) cholestatic (primarily elevated alkaline phosphatase), and (3) mixed (elevated AST, ALT, and alkaline phosphatase).

This patient developed ALF 10 days after initiating clarithromycin therapy. Other causes of ALF including viral, ischemic, autoimmune, and Wilson's disease and hemochromatosis were excluded. Echocardiography revealed a normal ejection fraction. The rise of AST and ALT to about 1,000 U/L each on second hospitalization day 2 preceded the hypotension from hemorrhagic shock which began 1 day later (AST and ALT rised to >6,000 U/L each; Table 1, Supplementary [Supplementary-material supplementary-material-1]). The initial rise in AST and ALT could not be attributed to shock liver, but the sharp rise in AST and ALT on day 3 might have been partly due to hepatic ischemia, from massive upper gastrointestinal hemorrhage associated with profound coagulopathy from hepatic failure. This may have contributed to finding hepatic necrosis at autopsy. Concomitant medications were unlikely causes of ALF because they were all chronically administered, except for prednisone which is not believed to cause ALF and has been previously used to treat one case of severe clarithromycin-induced hepatotoxicity [14]. Although renal insufficiency promotes clarithromycin-induced hepatotoxicity [15], the patient had normal renal function throughout the course of clarithromycin therapy.

Clarithromycin is synthesized by substituting a methoxy group for the C-6 hydroxyl group of erythromycin to improve oral bioavailability and gastrointestinal tolerance [16]. ALF has been reported secondary to other macrolides, especially erythromycin and roxithromycin [3, 4, 17, 18]. Clarithromycin is generally well tolerated, with dose reduction required in patients with renal insufficiency [15]. Clarithromycin-induced hepatotoxicity is attributed to (1) dose-related hepatotoxicity with elevated liver enzymes with ≥2 gm/day of clarithromycin in the elderly [19] and (2) possible idiosyncratic or hypersensitivity reactions with no evident dose-dependent relationship [11].

The epidemiology, presenting symptoms, signs, liver function tests, clarithromycin use, outcome, and confounding factors in the prior six cases of ALF associated with clarithromycin and the currently reported case are summarized in Table 2 [5–10]. Review of these 7 patients show that 5 were males (71%), on average were 41.7 ± 14.5 (SD) years old, and had onset of ALF 7.0 ± 3.4 days after onset of clarithromycin use. All patients had peak AST and ALT levels >1,000 U/L. Peak alkaline phosphatase levels ranged from 220–358 U/L, except 1 patient had a level of 1,095 U/L. Peak total bilirubin ranged from 1.7–33.4 mg/dL. All patients had predominantly hepatocellular injury, except that one patient had mixed hepatocellular and cholestatic injury [6]. All patients had significant coagulopathies (peak INR ≥1.9). In four patients having interpretable liver biopsies, performed premortem during ALF or postmortem, findings included moderate to massive hepatic necrosis in 3 and one each with bile duct proliferation, focal nodular regeneration, microvesicular steatosis, eosinophilic infiltration, or diffuse sinusoidal congestion.

Histopathologic analysis of the current patient's liver revealed diffuse hepatocellular necrosis, consistent with prior reported cases and idiosyncratic DILI as previously reported [20], but had no eosinophilic infiltrates characteristic of certain DILI pathology. Two prior patients also lacked eosinophilic infiltrates [5, 8]. Prominent eosinophilic infiltrates can suggest drug-induced hypersensitivity, but rarely occur in idiosyncratic drug reactions. No histopathologic findings are pathognomonic of DILI; a high index of clinical suspicion and compatible histopathologic findings are required for the diagnosis.

This review suffers from limitations of the 6 previously reported cases, including lack of liver biopsy (2) [7, 9], uninterpretable liver biopsy (1) [10], and administration of confounding potentially hepatotoxic drugs (3), including disulfiram [7], israpidine [8], or acetaminophen [5]. Other confounding variables included alcoholism (1) [7], heart failure (1) [9], and clarithromycin overdose (1) [10]. Also, hepatitis E, which can mimic DILI, [21] was not excluded in 4 prior cases [5, 7, 9, 10]. This work is also subject to limitations as one retrospectively reported case. The clinical picture of clarithromycin-induced hepatotoxicity might have been modified by terminal ischemic hepatitis from massive gastrointestinal bleeding during his last 24 hours of life that produced very high AST and ALT levels. However, the AST and ALT rise to about 1,000 U/L each one day earlier was associated with normal blood pressure readings and cannot be ascribed to shock liver/hepatic ischemia.

## 5. Conclusions

A 60-year-old nonalcoholic man, with normal baseline liver function tests, was admitted with diffuse abdominal pain and AST = 499 U/L and ALT = 539 U/L, 6 days after completing a 7-day course of clarithromycin 500 mg twice daily. AST and ALT each rose to ≥6,000 U/L two days later, accompanied by severe hepatic encephalopathy and coagulopathy. Extensive workup revealed no infectious, metabolic, or immunologic etiology of liver failure. The patient died soon thereafter from hemorrhagic shock from massive upper gastrointestinal bleeding and coagulopathy. Postmortem histopathologic hepatic examination revealed massive hepatocyte necrosis and diffuse sinusoidal congestion. Systematic review of 6 prior reported cases and this case of clarithromycin-associated ALF promotes further understanding of this syndrome.

## Figures and Tables

**Table 1:** Trend of liver function tests.

Laboratory value (normal laboratory range and units)	First hospitalization (16 days prior to 2^nd^ admission)	Day 1 second hospitalization (6 days after completing 7-day course of clarithromycin)	Day 2	Day 3	Day 4
Leukocyte count (3,500–10,100/*μ*L)	15,000/*µ*L	12,200/*µ*L			
Hemoglobin (13.5–17.0 g/dL)	13.6 g/dL	14.5 g/dL		5.5 g/dL	
Platelets (150,000–400,000/*μ*L)	209,000/*μ*L	129,000/*μ*L		35,000/*μ*L	
Aspartate aminotransferase (10–37 U/L)	39 U/L	499 U/L	1,119 U/L	9,470 U/L	10,820 U/L
Alanine aminotransferase (9–47 U/L)	72 U/L	539 U/L	1,110 U/L	6,420 U/L	7,210 U/L
Alkaline phosphatase (30–110 U/L)	55 U/L	127 U/L	171 U/L	358 U/L	202 U/L
Albumin (3.5–4.9 g/dL)	3.5 g/dL	3.4 g/dL	3.6 g/dL	2.9 g/dL	2.6 g/dL
Total bilirubin (0.3–1.2 mg/dL)	1.0 mg/dL	1.0 mg/dL	0.6 mg/dL	2.3 mg/dL	1.9 mg/dL
Direct bilirubin (0.0–0.4 mg/dL)	NA	NA	NA	1.8 mg/dL	1.3 mg/dL
INR (0.9–1.2)	NA	0.9		>9.0	>9.0

INR, international normalized ratio; NA, not applicable.

**Table 2:** Reported cases of fulminant liver failure associated with clarithromycin.

Author, year	Shaheen and Grimm, 1996 [5]	Christopher et al., 2002 [6]	Masia et al., 2002 [7]	Tietz et al., 2003 [8]	Albataineh and Siddiqui, 2007 [9]	Maggi et al., 2012 [10]	Current case
Age (in years), gender. Symptoms and signs on admission	25, M. Nausea, dark urine, and acholic stools. Afebrile, deep jaundice, and right upper quadrant tenderness.	40, F. Weakness, nausea, abdominal pain, and pyrexia. Had right upper quadrant tenderness.	47, M. Generalized maculopapular rash and malaise, high fever, blood pressure 90/60 mmHg, pulse 130/min, jaundice, and edematous extremities.	58, F. Nausea, jaundice, and diarrhea. Nontender liver palpable 2 cm below right costal margin, and bilateral trace ankle edema.	39, M. Right upper quadrant pain, recurrent emesis, and confusion 1 day after starting clarithromycin.	23, M. Diarrhea, vomiting, and fever starting after taking clarithromycin. Took overdose of clarithromycin 2 gm as initial dose.	60, M. Epigastric abdominal pain. Diffusely tender abdomen, hypoactive bowel sounds, and no stigmata of chronic liver disease.

Previous medical history	Noncontributory.	Hemodialysis for end-stage kidney disease, anemia, hypertension, pancreatitis, and pulmonary sarcoid without corticosteroid use. Status-post AV fistula and total abdominal hysterectomy, and cholecystectomy.	Receiving disulfiram for 1 month for chronic alcoholism. Received acetaminophen 1,500 mg/day together with clarithromycin.	Hypertension and mitral valve prolapse with regurgitation. Repair of lumbar disk herniation.	Congestive heart failure (ejection fraction of 10%), hypertension, and heavy alcohol use.	Schizoaffective psychosis treated with amisulpride, orphenadrine, and lithium carbonate. Two prior episodes of transient mild aminotransferase elevations.	Asthma treated with albuterol and budesonide, hypertension treated with amlodipine, metoprolol, and lisinopril, and hyperthyroidism treated with levothyroxine (all these medicines administered chronically)

Peak key liver laboratory values and imaging tests	AST 3,510 U/L ALT 4,790 U/L ALP 225 U/L TB 32 mg/dL, PT 30.9 s (no peripheral eosinophilia)	AST 2,255 U/L, ALT 1,974 U/L, ALP 1,095 U/L, TB 33.4 mg/dL, PT 21 s (eosinophil count: 430/*µ*L)	AST 1,149 U/L, ALT 2,603 U/L, ALP 240 U/L, TB 15.29 mg/dL, PT 21 s (no peripheral eosinophilia).	AST 23,166 U/L, ALT 13,853 U/L, ALP 258 U/L, TB 4.6 mg/dL, INR 5.7. Abdominal ultrasound: Enlarged and hyperechoic liver.	AST >6,000 U/L, ALT >4,000 U/L, PT 22 s. Abdominal ultrasound: Diffuse fatty liver, severe hepatomegaly.	AST 2,007 U/L, ALT 4,065 U/L, TB 10.0 mg/dL, INR 1.9	AST 10,820 U/L, ALT 7,210 U/L, ALP 358 U/L, TB 1.7 mg/dL, PT > 100 s. Abdominal ultrasound and CT: unremarkable liver. (No peripheral eosinophilia).

Reason for clarithromycin use; illness onset after starting clarithromycin therapy	Sinusitis, 9 days	Upper respiratory infection; 10 days	Pyrexia, odynophagia, and malaise for 7 days	Dry cough, fevers, and right lower lung infiltrate on chest radiograph; 4 days	Productive cough, dyspnea, pleuritic chest pain, left upper lobe infiltrate on chest radiograph. 1 day.	Sore throat. 8 days	Atypical chest pain, dyspnea, and productive cough. No pulmonic infiltrate on chest radiograph. 13 days after starting 7-day course of clarithromycin therapy.
Liver histologic findings	Explanted liver: massive hepatocyte necrosis, bile duct proliferation and areas of nodular regeneration.	Transjugular liver biopsy: moderate cholestasis, microvesicular steatosis, and eosinophilic infiltration.	No liver biopsy.	Diffuse, confluent necrosis involving centrilobular and midzonal areas.	Patient refused liver biopsy.	Autopsy showed marked cholestasis. Unable to fully assess microscopic hepatic structure due to advanced autolysis because autopsy performed 40 days after death.	At autopsy: diffuse sinusoidal congestion, massive hepatocyte necrosis, with occasional sparing of periportal hepatocytes.

Outcome	Underwent liver transplant after developing hepatic encephalopathy, and rising liver function tests. Died postoperatively from intracranial hemorrhage.	Developed progressive hepatic encephalopathy, and liver and pulmonary failure.	Transferred to liver transplant center for evaluation. However, rapidly died from septic shock.	Transferred to liver transplant center for evaluation. Developed severe leukocytosis, hepatic encephalopathy, very high aminotransferase levels and INR of 5.7. Recovered after stopping all medications.	After rapid worsening of liver failure when taking clarithromycin, liver function rapidly recovered towards normal when discontinuing clarithromycin.	Died from intracranial bleed (as shown by head CT). Bleeding related to coagulopathy from acute liver failure.	Rapidly expired from hemorrhagic shock from massive acute upper gastrointestinal bleeding, associated with profound coagulopathy. Planned liver biopsy could not be performed because of profound coagulopathy (see autopsy).

Confounding factors in liver injury	Took <5 gm/day of acetaminophen until 9 days before onset of symptoms. Serum acetaminophen level of zero on admission. Rarely drank alcohol. No serologic testing for hepatitis E.	Negative extensive workup for metabolic and infectious causes of liver disease. Normal ERCP findings. No recent acetaminophen use. No prior alcoholism. Extensive list of chronically used medications before instituting clarithromycin therapy.	Negative extensive workup for metabolic and infectious causes of liver disease. Disulfiram implicated as a cofactor in liver injury; disulfiram is a cytochrome P450 enzyme inhibitor which may increase clarithromycin levels. Taking acetaminophen 1,500 mg/day. No serologic testing for hepatitis E.	Recent trip to India. Social drinker of alcohol. Suffering from left-sided heart failure treated with atenolol. Also, receiving israpidine when developed liver failure.	Possible ischemic hepatitis from heart failure (ejection fraction = 10%); heavy alcohol use; started ceftriaxone concomitant with clarithromycin. Extensive workup found no other metabolic or infectious causes of acute liver failure. No serologic testing for hepatitis E.	Took four times normal initial dose of clarithromycin. Taking antipsychotic medications. Extensive workup found no other metabolic or infectious causes of acute liver failure. No serologic testing for hepatitis E. Liver histology at autopsy of limited value due to advanced autolysis because autopsy performed 40 days after death	Taking several other drugs for various diseases, but all of these drugs were chronically taken without prior toxicity. Extremely extensive workup found no other metabolic or infectious cause of acute liver failure. Had terminal hypotension from massive gastrointestinal hemorrhage in last 24 hrs (after having AST and ALT levels about 1,000 U/L each on the previous day).

AST, aspartate aminotransferase; ALT, alanine aminotransferase; ALP, alkaline phosphatase; TB, total bilirubin; PT, prothrombin time; INR, international normalized ratio; F, female; M, male; N/A,  not applicable.

## Supplementary Materials

Supplementary Materials Supplementary Table 1 reports the daily blood pressure recordings every morning on the first hospitalization and reports all the blood pressure recordings for the second hospitalization. This table demonstrates that the blood pressure throughout the first hospitalization was stable (and not hypotensive) and throughout the second hospitalization was stable (not hypotensive) until late in the evening of day 3 of the second hospitalization 16 hours before the patient's demise. These recordings demonstrate that the patient developed hypotension from hemorrhagic shock as a terminal event (associated with profound coagulopathy) after suffering acute liver failure associated with clarithromycin. Therefore, the hypotension did not directly cause the acute liver failure but was a consequence of the acute liver failure.
