# Supplementary material for: Clarithromycin-Associated Acute Liver Failure Leading to Fatal, Massive Upper Gastrointestinal Hemorrhage from Profound Coagulopathy: Case Report and Systematic Literature Review
Source: Case Reports Hepatol. 2020 Feb 18;2020:2135239. doi: 10.1155/2020/2135239 (PMC7049424; doi:10.1155/2020/2135239)
Supplement: Supplementary Materials — Supplementary Table 1 reports the daily blood pressure recordings every morning on the first hospitalization and reports all the blood pressure recordings for the second hospitalization. This table demonstrates that the blood pressure throughout the first hospitalization was stable (and not hypotensive) and throughout the second hospitalization was stable (not hypotensive) until late in the evening of day 3 of the second hospitalization 16 hours before the patient's demise. These recordings demonstrate that the patient developed hypotension from hemorrhagic shock as a terminal event (associated with profound coagulopathy) after suffering acute liver failure associated with clarithromycin. Therefore, the hypotension did not directly cause the acute liver failure but was a consequence of the acute liver failure. [file 2135239.f1.docx]

| Supplementary Table 1. Blood Pressure measurements throughout patient hospitalizations 1 and 2. | | | | | | | | | |
| --- | --- | --- | --- | --- | --- | --- | --- | --- | --- |
| Day of first admission | Blood pressure (mmHg) |  |  |  |  |  |  |  |  |
| Day 1 | 154/102 |  |  |  |  |  |  |  |  |
| Day 2 | 154/85 |  |  |  |  |  |  |  |  |
| Day 3 | 150/102 |  |  |  |  |  |  |  |  |
| Day 4 | 163/98 |  |  |  |  |  |  |  |  |
| Day 5 | 147/90 |  |  |  |  |  |  |  |  |
| Day 6 | 158/88 |  |  |  |  |  |  |  |  |
| Day 7 | 171/99 |  |  |  |  |  |  |  |  |
| Day 8 | 145/86 |  |  |  |  |  |  |  |  |
| Day 9 | 149/99 |  |  |  |  |  |  |  |  |
| Day 10 | 131/84 |  |  |  |  |  |  |  |  |
| Day 11 | 116/74 |  |  |  |  |  |  |  |  |
| Day of second admission (10 days after first hospital discharge ) | | | | | | | | | |
| Day 1 | 140/92 | 140/85 | 149/96 | 147/92 | 143/92 | 161/102 | 158/95 |  |  |
| Day 2 | 161/98 | 162/103 | 161/91 | 169/102 | 176/103 | 164/100 | 143/92 |  |  |
| Day 3 | 156/98 | 142/89 | 119/74 | 116/92 | 94/64 | 132/42 | 110/49 | 122/44 | 103/39 |
| Day 4 | 94/40 | 88/38 | 61/31 | 77/43 | 86/47 | 71/39 | 79/43 | 86/47 | 71/39 |
